# From symptoms to subjective and bodily experiences: the contribution of the *Psychodynamic Diagnostic Manual* (PDM-2) to diagnosis and treatment monitoring in eating disorders

**DOI:** 10.1007/s40519-023-01562-3

**Published:** 2023-03-30

**Authors:** Marta Mirabella, Laura Muzi, Anna Franco, Alessia Urgese, Michele A. Rugo, Claudia Mazzeschi, Anna Maria Speranza, Nancy McWilliams, Vittorio Lingiardi

**Affiliations:** 1 Department of Dynamic and Clinical Psychology, and Health Studies, Sapienza University of Rome, Rome, Italy; 2 Department of Philosophy, Social Sciences, Humanities and Education, University of Perugia, Perugia, Italy; 3 Eating Disorder Clinic “Residenza Gruber”, Bologna, Italy; 4 Graduate School of Applied and Professional Psychology, Rutgers University, Lambertville, NJ USA

**Keywords:** Eating disorders, Diagnosis, *Psychodynamic Diagnostic Manual-2*, Subjective experience, Process-outcome research, Clinical utility

## Abstract

**Purpose:**

Atheoretical and descriptive conceptualizations of eating disorders (EDs) have faced substantial criticism due to their limited ability to assess patients’ subjective characteristics and experiences, as needed to determine the most appropriate treatment options. The present article provides an overview of the clinical and empirical literature supporting the potential contribution of the *Psychodynamic Diagnostic Manual* (PDM-2) to both diagnostic assessment and treatment monitoring.

**Methods:**

Following a discussion of the most relevant shortcomings of current diagnostic models of EDs and a description of the rationale and structure of the PDM-2, evidence supporting the core PDM-2 dimensions of ED patients’ subjective experiences (i.e., affective states, cognitive processes, relational patterns, somatic/bodily experiences and states) are examined, alongside their relevance to ED diagnosis and treatment.

**Results:**

Overall, the reviewed studies support the diagnostic importance of these patterns of subjective experiences in EDs, highlighting their potential role as either predisposing or maintaining factors to target in psychotherapy. A growing body of multidisciplinary evidence also shows that bodily and somatic experiences are central to the diagnosis and clinical management of ED patients. Moreover, there is evidence that a PDM-based assessment may enable closer monitoring of patient progress during treatment, with regard to both subjective experiences and symptom patterns.

**Conclusions:**

The study suggests that current diagnostic frameworks for EDs would benefit from the addition of a person-centered perspective that considers not only symptoms, but also patients’ full range of functioning—including their deep and surface-level emotional, cognitive, interpersonal, and social patterns—to improve patient-tailored interventions.

**Level of evidence:**

Level V, narrative review.

## Introduction

Eating disorders (EDs), including anorexia nervosa (AN), bulimia nervosa (BN), binge eating disorder (BED), and otherwise specified feeding and eating disorders (OSFED), are complex mental illnesses associated with significant clinical impairments, increased mortality, decreased quality of life, and socioeconomic costs [1, 2]. Furthermore, previous studies have shown that, relative to patients with other mental disorders, ED patients are more likely to experience treatment failure [3], ranging from dropout [4] to common relapse [5]. In this perspective, the definition and classification of EDs may have pivotal implications for both scientific research and ED treatment planning. At an empirical level, some authors [6] have claimed that the tendency to adopt a primarily rigid, categorical, and symptom-oriented definition of ED in empirical studies has likely hindered the progression of clinical and scientific knowledge about the etiology, onset, course, maintenance, clinical presentation, and recovery rate of EDs. At a clinical level, most practice guidelines (e.g., [7, 8]) agree that there should be a continuum of care for ED patients, with psychosocial interventions chosen according to a comprehensive understanding of patients’ individual characteristics and differences.

From a diagnostic standpoint, current conceptualizations of EDs have been subject to several criticisms [9]. Descriptive, atheoretical, and symptom-oriented approaches, such as those promoted in the fifth revised edition of the *Diagnostic and Statistical Manual of Mental Disorders* (DSM-5-TR) [10] and the eleventh edition of the *International Classification of Diseases* (ICD-11) [11], suffer from high temporal instability and a lack of construct validity within the identified core symptoms. This is due to “migration” between categorical ED diagnoses, which show significant overlap in their symptomatic behaviors, impaired interpersonal functioning [12], and comorbid disorders [13]. Furthermore, the percentage of ED patients falling within the highly heterogeneous OSFED and “unspecified feeding or eating disorder” (UFED) categories [14] has been estimated as 53% in studies conducted prior to the publication of the DSM-5, and 25–26% more recently [15–17]. Both the DSM-5 and the ICD-11 seem to overlook the high individual variability within specific ED diagnoses, in terms of symptom severity, personality characteristics, cognitive styles, and medical or psychiatric comorbidities [18]. Limitations can also be found in the novel *Hierarchical Taxonomy of Psychopathology* (HiTOP), which includes EDs on its internalizing and somatoform spectra, known as the emotional dysfunction superspectrum [19, 20]. Despite the clinical utility of the HiTOP’s dimensional approach and hypothesized core symptoms of EDs (e.g., body dissatisfaction, cognitive restraint, negative attitudes toward obesity) [21], there is a paucity of research to support its validity. Moreover, some authors have cast doubt on the model’s grouping of EDs within somatoform disorders, claiming instead that these should be considered within a separate structural dimension [22].

Other ED models have been proposed [23], including the *transdiagnostic maintenance model of EDs* [24], which assumes that the core feature of all EDs is an overvaluation of control over one’s body shape, weight, and eating. In contrast, the empirically based *three-dimensional model* (TDM) of EDs [25] hypothesizes the pivotal role of binge eating, drive for thinness, and fear of fatness/inappropriate compensatory behaviors. Other proposed models, such as the *cognitive-interpersonal maintenance model* of AN [26], the *dual-pathway model* for BN [27], and the *interpersonal model* of BED [28], primarily focus on specific diagnoses. Although the theoretical underpinnings of these conceptual models have received support, they are often disentangled from nosological approaches to ED diagnoses, and they have rarely been applied to support the development of ED interventions [23].

These observations have relevant therapeutic implications. First and foremost, the *raison d’etre* of any diagnostic system is its utility in clinical settings [29]. Despite their unquestionable advantages for empirical and epidemiological purposes, most diagnostic models of EDs do not offer therapeutic guidelines or recommendations for case formulation and treatment planning, for use by psychotherapists treating ED patients. Additionally, the DSM-5 and ICD-11 severity specifiers for EDs have not emerged as reliable predictors of patients’ responses to treatment, dropout rates, recovery rates, or outcomes following different psychosocial interventions [30, 31]. Second, these approaches are not intended to serve as reliable assessment tools for regular, quantitative evaluations of symptomatic change throughout treatment (or measures at the initiation or termination of treatment); thus, they do not necessarily support treatment monitoring [32, 33]. Conversely, a growing body of evidence suggests that outcome monitoring might inform individualized care for EDs and prevent treatment non-response or failure [34].

Finally, as descriptive and atheoretical approaches to EDs, the DSM-5 and ICD-11 are non-inferential, aimed at removing “bias” from the psychodynamic tradition by disregarding the subjective experiences of both patients and clinicians. In this regard, some authors have even described the subjective experiences of patients as an “obstacle” [35]. However, most practitioners in the ED field begin their psychological evaluations by trying to understand the meaning and function of ED patients’ difficulties in the larger context of their personality and overall functioning. This lack of accounting for the internal experiences of ED patients, and the related inability of the most common diagnostic approaches to apply this information in support of patient-tailored therapeutic interventions, might represent a significant weakness of current conceptualizations of EDs.

## The present study

In light of the aforementioned shortcomings of current ED models, the present article provides an overview of the clinical literature and empirical research supporting the relevance of the complementary, psychodynamic-oriented approach to EDs proposed in the second edition of the *Psychodynamic Diagnostic Manual* (PDM-2) [36]. The main aim of the work is to outline the clinical utility and empirical validity of the PDM-2 for the diagnosis and treatment monitoring of EDs. To this end, the PDM-2 conceptual model is first introduced, to provide a theoretical framework for the key research questions:

- *Question (1)*: Is the PDM-2 approach supported by empirical research?
- *Question (2):* Are the key areas of patients’ subjective experiences, as indicated by the PDM-2, relevant to ED diagnosis and treatment?
- *Question (3):* Do bodily experiences and feelings about the body contribute to ED clinical presentations?

### Beyond symptoms: the *Psychodynamic Diagnostic Manual* (PDM-2) model

The *Psychodynamic Diagnostic Manual* (PDM-2) [36] offers a complementary perspective to the descriptive systems of the DSM, ICD, and HiTOP, promoting a diagnostic approach that considers not only symptoms, but also idiographic, subjective characteristics and psychological functioning in different life stages. Accordingly, the PDM-2 approach supports clinicians in their efforts to: understand the depth and surface of their patients’ emotional, cognitive, interpersonal, and social patterns; make “clinically meaningful” and empirically grounded diagnoses; take developmental perspectives into account; and integrate other branches of knowledge and theoretical traditions into their diagnostic process. Thus, it aspires to provide a “taxonomy of people,” rather than a “taxonomy of disorders,” highlighting the importance of considering who a patient is, rather than what a patient has [37, 38], in order to enrich case formulations and guide patient-tailored treatment planning.

The PDM-2 is divided into sections pertaining to: “Adulthood,” “Adolescence” (i.e., ages 12–18 years), “Childhood” (i.e., ages 4–11 years), “Infancy and Early Childhood” (i.e., ages 0–3 years), “Later Life,” and “Assessment and Clinical Illustrations” (including the measure derived from the manual) [39]. In each section, the conceptual framework is structured across three axes that systematically describe healthy and disordered *levels of personality organization* and *personality styles/syndromes* (P Axis); individual profiles of *mental functioning* (e.g., patterns of relating to others, comprehending and expressing feelings, coping with stress and anxiety, regulating impulses, observing one’s own emotions and behaviors, and forming moral judgments) (M Axis); and symptom patterns, including individual differences in personal, *subjective experiences of symptoms* and the related experiences of treating clinicians (S Axis). To achieve a holistic diagnosis, all three axes must be evaluated in each patient. The order in which these axes are evaluated varies according to the patient’s life stage. In adults, personality (P Axis) is evaluated prior to mental functioning (M Axis) and symptomatic patterns (S Axis), because this dimension is quite stable and usually demands the primary clinical focus [3]. Lastly, PDM-2 diagnoses are “prototypical”—that is, the descriptions are best understood as “ideals” that an individual may approximate to a greater or lesser extent, and not as distinct categories based on a list of symptoms and signs. Evidence suggests that, when making diagnoses, clinicians tend to think in terms of prototypes, even as they speak in terms of categories [40]. Furthermore, while referring to DSM and ICD diagnostic labels, the PDM-2 outlines patients’ subjective experiences related to these labels.

The manual describes EDs in adult patients (specifically AN and BN) within the “Specific Symptom Disorders” section of the S Axis. In describing these disorders, the manual aims at preserving and reinforcing the primacy of patients’ subjective experiences of symptom patterns, in line with a psychodynamic approach. Of note, the S Axis works jointly with the P and M Axes to generate a comprehensive representation of the psychological and/or psychopathological functioning of the whole person. Thus, the S Axis provides only one of three crucial perspectives and assists clinicians in creating a multifaceted diagnostic profile of the patient, to determine the best treatment options [41]. With respect to personality features in EDs, the manual highlights the relevance of three empirically-based personality configurations that broadly correspond to: (a) an *underregulated* subtype, characterized by patterns of impulsive behavior and affective lability/instability, borderline and bulimic features, and feelings of emptiness and emotional hunger [42]; (b) an *overregulated* subtype, characterized by inhibition and a restricted behavioral/affective presentation; schizoid, avoidant, and obsessive–compulsive features; and anorexic symptoms [43]; and (c) a *high-functioning/perfectionistic* subtype, characterized by normative levels of personality functioning and less severe ED psychopathology [44]. These personality profiles have been confirmed by a substantial body of research using different assessment tools with both single diagnostic and mixed ED samples [45–49].

As detailed in the following paragraphs, the S Axis describes four domains of ED patients’ subjective experiences. This first domain pertains to the most common *affective states* associated with EDs, such as feelings of being starved for care and affection, guilt, weakness, anger, unworthiness, emptiness, fear of abandonment, and loss of control. The second domain describes relevant *cognitive patterns*, which include rigid thinking and perceptual distortions of one’s own body or body image; and preoccupation with being devalued, inadequate, incompetent, or unloved. The third area focuses on *bodily and somatic states*, which often involve the effect on the real body of mental conflict and impairments in differentiating between mental and somatic states. The fourth domain pertains to the most recurrent relational patterns, including difficulties with emotional intimacy and a pervasive need for control and perfectionism or, conversely, frequent abandonment or engulfment anxieties [50]. Lastly, the S Axis also considers the subjective experiences of the treating clinician (i.e., the therapist’s emotional response or countertransference patterns). The following section presents a review of the clinical and empirical literature supporting these patterns of subjective experiences in ED patients, highlighting their potential relevance for the identification of factors to target and monitor in psychotherapy.

### Is the PDM-2 approach supported by empirical research?

One of the main goals of the PDM-2 is to better integrate the diagnostic process with clinical practice and empirical research. Several clinicians and researchers have sought to counteract the widespread belief that psychodynamic diagnosis and therapeutic approaches lack empirical support, especially with respect to EDs [51, 52]. In line with this, the PDM-2 aims to be based on empirical evidence and to be empirically tested in its fundamental assumptions and principles [53].

In this regard, several efforts have been made to empirically refine the manual. The first such effort involved the development of valid and reliable PDM-based assessment tools, and particularly the Psychodiagnostic Chart, which is now in its second version (PDC-2) [54]. The PDC-2 is a clinician-rated coding tool that allows practitioners to combine DSM and ICD labels with PDM-derived models of personality organization, overall mental functioning, patterns of patients’ subjective experiences, and other salient psychological, cultural, and contextual variables [55]. The current version is based on 10 years of field testing and evidence gathered from practitioners of various theoretical orientations [56, 57], and several studies have demonstrated its good reliability and construct validity [39, 55, 58]. Furthermore, there are parallel forms of the PDC-2 tailored to the different age groups considered in the PDM-2, making the tool highly applicable across the entire life span [59], as well as across different clinical and research settings [39, 55, 57, 60–62].

The development of this PDM-based assessment tool has had pivotal implications for treatment monitoring. Most available measures for ED outcome monitoring primarily assess DSM symptoms or, conversely, patients’ general functioning in daily life (e.g., occupational and social role functioning), and show limited clinical utility for patients with severe EDs, who require more intensive levels of care [34, 63]. Additionally, the common ego-syntonic and reinforcing nature of several ED symptoms, as well as patients’ lack of insight or even denial of the illness, may limit the ability of self-report measures to detect changes in symptoms throughout treatment. The PDC-2 has been used extensively to evaluate patients’ treatment progress in single case studies, revealing its validity and utility as an effective tool to explore both symptom changes and “structural” changes in personality organization over time, according to patient narratives [64, 65].

Both single cases and quantitative studies involving different clinical populations have shown that the PDC-2 supports the assessment of key dimensions of psychological functioning underlying observable symptoms, including defense mechanisms, mental functioning capacities, and personality styles or types [39, 55, 64–66]. For instance, the single case study by Tanzilli et al. [65] employed the PDC-2 to obtain a comprehensive picture of an adolescent patient with major depressive disorder through the lens of mental functioning and levels of personality organization. Specifically, a borderline personality organization was found to be associated with an impaired ability to engage in stable and satisfying intimate relationships and to regulate self-esteem, which, in turn, impacted on the psychodynamic psychotherapy outcome. Another single case [64] applied the PDC-2 to the Adult Attachment Interview [67] in an adult patient with an anxiety disorder, showing the relevance of impaired reflective functioning and relationship skills, as well as the severity of anxiety symptoms, in determining therapeutic change.

From a quantitative perspective, another study applied the PDC-2 to assess levels of personality organization in a sample of 88 help-seeking patients with mixed diagnoses, finding that this variable was related to other clinically relevant psychodynamic variables (e.g., defensive functioning, object relations) [55]. Finally, a recent investigation applied the PDC-2 to examine whether the domains assessed by the PDM-2 have relevant implications for determining the responses of ED patients to a psychodynamic-oriented residential treatment program. The findings showed that, over and above the DSM-5 ED diagnoses of AN or BN, higher levels of personality organization and less severe personality pathology, in addition to higher mentalizing capacity, identity integration, and self-coherence, were related to better therapeutic outcomes [44].

Finally, previous empirical studies have also shown the perceived utility of the PDM-2 in clinical practice compared to other diagnostic systems (e.g., DSM, ICD). Gordon [57] found that diverse psychotherapists evaluated the PDM approach favorably, regardless of their theoretical orientation, emphasizing the value of its jargon-free language and its ability to support non-psychodynamic clinicians in their efforts to formulate a clinically relevant diagnosis. Other studies have found that both experienced and trainee clinicians rate the PDM model as the easiest and the most useful for assessing personality functioning and disorders, compared to other diagnostic approaches. Notably, participants reported that the PDM-2 model provided a comprehensive and in-depth picture of their patients [68–70].

### Are the key areas of patients’ subjective experiences, as indicated by the PDM-2, relevant to ED diagnosis and treatment?

As previously mentioned, the S Axis takes as its starting point the DSM-5 and ICD-11 diagnostic criteria for EDs, while also integrating idiographic patterns (i.e., affective states, cognitive processes, relational patterns, somatic states) that shape patients’ presenting symptomatology [41], and common therapist emotional responses (i.e., countertranference patterns).

#### Affective states

In line with the growing literature on affective dysfunction in EDs [e.g., 71, 72], the PDM-2 strongly emphasizes ED patients’ difficulties in affective functioning and emotion regulation, which have been observed since the earliest descriptions of the disorders. Charles Lasègue described a patient with AN as a young woman who “suffers from some emotions she avows or conceals” [73]. A century later, Hilde Bruch [74] postulated that women with AN have an underlying deficiency in the identification of emotional states and responses. More contemporary psychodynamic views of affective dysregulation in EDs posit that primary caregivers act as useful and essential leaders for their children’s scouting of reality during feeding times, through affect mirroring. Thus, impairments in affective functioning may arise from failure in the primary parental holding system, making the subjective experience of the child unbearable and overwhelming, and creating “indigestible” affective states [74].

Empirical evidence mainly supports the view that disordered eating behaviors and ED symptoms are attempts to downregulate negative affect and undesirable mood states. First, several studies have highlighted that higher levels of depressive symptoms and anhedonia may predict ED symptom severity and treatment outcomes [75, 76]. Second, other investigations have found that emotional dysregulation is closely associated with eating pathology—at both a symptom and a disorder level—irrespective of the specific ED diagnosis [77]. Research has also shown a common comorbidity between EDs and the affective features of borderline personality disorder (BPD). For instance, De Paoli et al. [78] found that body dissatisfaction was related to the BPD symptom of affective instability and emotion dysregulation. Similarly, affective instability has been found as the most relevant BPD symptom in ED patients compared to controls, with a larger effect size than that of other features (e.g., abandonment avoidance, suicidal behaviors) [72]. Explorations of psychopathological traits in ED patients have noted the significant presence of anger, especially in BN patients [79], as well as self-criticism, self-hostility, guilt, and shame [80]. Overall, these findings suggest that EDs may be conceptualized as paradoxical expressions of overwhelming emotional pain in circumstances where the ability to think about painful mind states is missing [81].

#### Cognitive patterns

With respect to cognitive patterns and thought processes, EDs may span the spectrum from neurosis to psychosis, though most manifestations are closely related to personality disorders. In more severe cases, patients’ reality testing may be impaired, leading to extreme rigid thinking and severe perceptual body distortions [82]. Paradoxically, some may even feel “subjectively” better as their health “objectively” worsens, due to their increasing physical alignment with their thin ideal [41]. Other common cognitive patterns include a focus on being young, “little,” childlike, and innocent, implying an unconscious wish to avoid puberty and adulthood, as well as high levels of perfectionism (associated with narcissism), and an excessive interest in body image checking. Since the 1970s, theoretical accounts of eating pathology have emphasized perfectionism and so-called “maturity fears” (included in several widely used self-report measures of ED pathology, such as the Eating Disorder Inventory-3) [83]. Bruch’s theory posits that AN patients display a perfectionistic drive to achieve and a tendency to conform to external standards of success, which, in combination, may trigger an intense pursuit of societal standards of thinness. Furthermore, weight loss (and its result of a childlike figure) has been conceptualized as an attempt to return to the security of childhood, triggered by the challenges of adolescence (see also [84]).

Some empirical research has found that perfectionistic traits are significant predictors of ED symptom severity and a maintaining factor for partial or full-blown EDs over time, even at 10-year [85], 12-year [86], and 30-year follow-ups [87]. Perfectionism has also been shown to predict worse therapeutic outcomes at a 16-year follow-up [88]. Furthermore, some longitudinal studies have shown that greater maturity fears at baseline predict a higher drive for thinness and more bulimic symptoms at 10-year, 20-year, and 30-year follow-ups [87], as well as worse AN outcomes at a 20-year follow-up [89]. Finally, body checking (i.e., scrutinizing one’s body in a mirror, checking the fit of clothes, measuring body parts) has emerged as both a maintaining factor of eating pathologies and a trans-diagnostic treatment target [90]. While self-referential ruminations about body shape and size may emerge as a biological consequence of starvation, for most ED patients, these cognitive patterns leave little room for genuine expressions of emotional and relational drives and needs. Instead, they reflect an overreliance on “emotional escapism,” which creates suffering for the patient and results in poor treatment outcomes.

#### Interpersonal patterns

As outlined in the PDM-2, ED patients tend to adopt maladaptive interpersonal behaviors that serve to regulate emotions, avoid confrontation, and manage negative experiences [71]. Commonly, AN patients tend to show high levels of social anxiety and a need for control and perfectionism in interpersonal relationships, while BN patients tend to crave love and fear abandonment, yet struggle with feelings of anger, intrusion, and anxiety in their relationships; accordingly, their relationships are frequently chaotic and unstable. From a psychodynamic perspective, EDs are hypothesized to be related to deficits in the interactive regulation of emotional states, due to early interpersonal and familial patterns characterized by entanglement and emotional neglect [91]. More specifically, research has shown that attachment insecurity may be pivotal for determining the onset, maintenance, and course of eating pathologies [92]. For instance, empirical studies have found that maladaptive perfectionism, hypermentalization, and difficulties in emotion regulation mediate the effects of insecure attachment on ED symptomatology; furthermore, maladaptive affect regulation associated with attachment insecurity may play a key role in the expression and maintenance of disordered eating and ED symptoms [93]. Overall, research suggests that attachment-related internal working models, which have their roots in early caregiving relationships, might lead to difficulties in affect regulation, perfectionism, and adult attachment insecurity that, in turn, may determine higher vulnerability to ED symptoms, including body dissatisfaction [92].

#### Therapist emotional responses

According to empirical findings [94–96], the S Axis suggests that ED patients tend to evoke strong and intense emotional reactions in therapists (i.e., countertransference patterns) that are often unique in their affective quality (involving, e.g., anger, hatred, despair, commiseration, grief, or love) and difficult to manage in psychotherapy. More specifically, therapists tend to report more disorganized yet parental/protective feelings toward BN patients, and more overwhelmed and overinvolved feelings toward AN patients [97]. However, therapists’ countertransference patterns may also be strongly influenced by trans-diagnostic variables. For instance, ED patients with higher levels of personality impairment and/or personality disorders tend to evoke stronger feelings of inadequacy, disorganization, and disengagement, in addition to lower positive reactions, in their treating clinicians [96, 97].

### Do bodily experiences and feelings about the body contribute to ED clinical presentations?

As outlined in the last domain of the S Axis, ED patients tend to subjectively experience a wide range of bodily sensations and body image–related symptoms that are primarily associated with negative and/or altered perceptions, thoughts, feelings, attitudes, and beliefs toward the body. Within the multidimensional construct of body image, the most investigated facets in EDs are distorted body image, body shape/weight dissatisfaction, discomfort and detachment feelings toward one’s body, and specific concerns about particular body parts, shapes or functions, which may induce avoidance or checking attitudes [98]. Additionally, ED patients may report feeling that their body changes continuously and unpredictably [99, 100]. ED symptoms such as starvation, thinness, and binge eating may be underpinned by an underlying set of values triggered by a disturbed body experience [101]. This may relate to experiences of pathological failure in early maternal responsivity and maternal impingement, resulting in a mind–body split and what has been defined by Bach [102] as a “disembodied self.” Indeed, an infant’s first experiences of being touched and held by a caregiver have been hypothesized to trigger the formation of a psychic space in which mental representations are held [103]. In this perspective, physical touch from a caregiver may encourage the child’s developing capacity for psychic containment. Krystal [104] suggested that affects are initially experienced as bodily sensations, before they are progressively differentiated into psychic states. Therefore, when subjects experience insecure and/or traumatic attachment relationships, their mind–body connection and capacity to regulate emotions may be compromised, forcing self-regulation to be performed in a more concrete and stereotypic manner.

Consistent with Bruch’s [74] hypothesis that ED patients demonstrate an “interoceptive problem”—that is, difficulty distinguishing between inside and outside and between self and other—the S Axis suggests that, for many ED patients, food and the body become the primary targets of self-expression. Specifically, in the face of unprocessed trauma and emotions, the body may become the tool with which individuals with EDs desperately attempt to gain mastery and control over their feelings [101]. For instance, they may misread the somatic sensation of hunger as a subjective feeling of emptiness or a desire for emotional bonding; alternatively, binge eating or elimination behaviors might be psychopathological correlates of underlying identity diffusion or dissociation. Furthermore, painful self-perceptions or negative affects and emotional states may be primarily expressed through extreme body aversion, together with the mistaken belief that altering the body will bring about higher levels of self-acceptance, confidence, and agency.

Previous empirical research has provided support for the relevance of disturbances in body image and bodily experiences in ED patients. First and foremost, a recent review of the main conceptual models of EDs and disordered eating showed that negative body image constructs (e.g., body weight and shape concerns, body image disturbance, body dissatisfaction, body uneasiness) were common risk factors (over and above ED categories in the DSM) [8] and potential trans-diagnostic targets for therapeutic interventions. In this perspective, Abbate-Daga et al. [51] found that patients with early-onset AN, compared to those with late-onset AN, showed higher levels of body uneasiness and dissatisfaction. Similarly, Carter et al. [105] found that concerns about body shape and weight in AN patients predicted relapse rates 6–17 months after discharge. Moreover, Bijsterbosch [106] found that body avoidance and body-checking dimensions predicted the maintenance of AN over time. Other studies have found that higher levels of body dissatisfaction are associated with compensatory behaviors and fear of weight gain, and predict greater overall ED psychopathology and BN symptoms [29].

Another field of research on the bodily experiences of ED patients concerns common disturbances in their perception or cognitive interpretation of somatic, body-based stimuli (e.g., hunger, fullness, satiety) [107, 108]. Confusion about these somatic states may, in turn, explain some disordered eating behaviors, such as meal skipping, food restriction, and binging or overeating. Interoceptive deficits and impaired mind–body differentiation may also predict severe difficulties in the ability to regulate, symbolize, and express affective states, which may instead be experienced as somatic issues or problems. Additionally, empirical evidence has revealed deficits in ED patients’ somatosensory perception [109], with consequences for their mental representations of the body (i.e., abstract and perceptual representations of body characteristics, referring to shape, size of body parts, position of body parts in space, and the integration of different body parts). Specifically, ED patients may experience altered bodily attitudes (i.e., thinking and/or imagining themselves as fat) and distortions in their visual [109], haptic [110], and tactile perceptions of the body as well as affordance perception/bodily action [111]. Additionally, rigid cognitive thoughts may determine a bias in visuospatial ability (i.e., estimation accuracy [111]), which may lead to a perceptual overestimation of body size and shape [110, 111]. Research has shown that such impairments in somatosensory perception have clinical relevance for the maintenance and course of EDs [109].

All of the abovementioned studies, stemming from different branches of psycho(patho)logical research, support the PDM-2’s emphasis on ED patients’ somatic and bodily experiences as a clinically relevant dimension of their subjective experiences. This implies the need for an in-depth evaluation of ED patients’ experiences and perceptions of their body and bodily symptoms to inform patient-tailored interventions. As eating pathologies may be viewed as disorders of self-regulation that center on the body [101], ED patients’ somatic experiences might be particularly relevant to their treatment. Accordingly, the body must be considered a psychotherapeutic tool that can help therapists connect with and respond to patients’ “unformulated experiences,” through the identification and containment of bodily sensations and affects, and their articulation in words. Moreover, therapists must use their own bodies as a medium for picking up non-verbal information from ED patients. In this way, they may better understand patients’ bodily experiences [101], which may contribute to strengthening the patient–therapist relationship—one of the most robust predictors of therapy outcome [112].

## Conclusions and future directions

In a meditation on “what we diagnose,” Karl Jaspers [113] described that every mental disorder “corresponds to the psychic level of the individual who showed it” (p. 14), and that every diagnosis should be typological and multidimensional, drawing on in-depth knowledge of the patient’s subjective characteristics (e.g., personality traits, affective states, interpersonal patterns, and other relevant domains of mental and psychological functioning). At the same time, he outlined that “every diagnostic schema must remain a torment for the scientist.” This “torment,” which is particularly relevant in the treatment of eating pathologies, may be understood as the tension that is inherent in every diagnostic process—that is, the tension of integrating complex clinical phenomena (representing a functional understanding) and reliable diagnostic criteria (representing a descriptive understanding) into a nomothetic understanding, and integrating idiographic knowledge to emphasize both individual subjective variations and commonalities.

A growing number of clinicians and researchers in the field of EDs are deeply aware of the need to overcome the limitations of atheoretical descriptions of psychological syndromes. Instead, they are turning to embrace more clinically relevant and person-centered conceptual and diagnostic models, which are sufficiently psychologically rich to guide effective treatment planning (especially when psychotherapy is among the recommended interventions). In this perspective, the PDM-2 aims at offering a psychodynamic diagnostic framework for EDs that emphasizes and “regulates” the subjectivity of both patients and clinicians, based on the assumption that every ED patient has a unique and individual potential, treatment need, and response to treatment [10].

The PDM-2 attempts to complement the ocularcentrism and nosographism of current diagnostic conceptualizations of EDs [114], which may have meaningful implications for research on ED therapy and outcomes. Specifically, the literature on the effectiveness of psychodynamic psychotherapy for EDs is growing but still limited, despite reporting promising preliminary findings [115]. It remains difficult for researchers to properly capture and monitor therapeutic change in patients’ subjective concerns over time; rather, research generally focuses on changes in observable symptoms, or the restoration of weight and body mass index [51]). However, psychodynamic psychotherapies aim at reducing patients’ perfectionistic attitudes, improving patients’ sense of security and willingness to engage in interpersonal relationships, enhancing patients’ ability to self-reflect (i.e., mentalize), and reducing patients’ self-destructive relationships and behaviors. Thus, the PDM-2 model for EDs has the potential to enhance research on treatment efficacy and outcome monitoring [116] by considering changes in patients’ unique self-experiences and the meaning/function of their symptoms over time.

Some limitations and future directions should be acknowledged. First, PDM-based research on the diagnosis and treatment of EDs is still in its infancy. Future studies should apply the PDC-2 assessment tool to ED samples across different therapeutic settings, to empirically investigate the reliability, construct validity, and practical use of the tool’s dimensions and scales. Such research would also benefit from the addition of other psychodynamic-grounded empirical measures (e.g., the Shedler–Westen Assessment Procedure-200 [117], which has been previously applied to ED samples) [e.g., 44, 47, 117]. Furthermore, as the PDM-2 aims at overcoming the limitations of the DSM-5, ICD-11, and HiTOP through the addition of a person-centered and clinically useful perspective on EDs, future investigations should systematically compare the clinical utility of its approach with these other diagnostic models, in practice. Finally, despite a growing body of evidence supporting the relevance of the four domains of ED patients’ subjective experiences, as indicated by the PDM-2 S Axis (i.e., affective states, cognitive patterns, somatic and bodily experiences, interpersonal patterns), as well as the potential role played by the therapist’s subjective experiences or emotional responses, more research is needed to explore how these dimensions may interact with ED patients’ personality features and overall mental functioning, in determining the symptomatic presentation and clinical course of EDs.
